# Protection against H1N1 influenza challenge by a DNA vaccine expressing H3/H1 subtype hemagglutinin combined with MHC class II-restricted epitopes

**DOI:** 10.1186/1743-422X-7-363

**Published:** 2010-12-07

**Authors:** Lei Tan, Huijun Lu, Dan Zhang, Mingyao Tian, Bo Hu, Zhuoyue Wang, Ningyi Jin

**Affiliations:** 1 College of Animal Science and Veterinary Medicine, Jilin University, Changchun 130062, PR China; 2 Genetic Engineering Laboratory, Academy of Military Medical Sciences, Changchun 130062, PR China

## Abstract

**Background:**

Multiple subtypes of avian influenza viruses have crossed the species barrier to infect humans and have the potential to cause a pandemic. Therefore, new influenza vaccines to prevent the co-existence of multiple subtypes within a host and cross-species transmission of influenza are urgently needed.

**Methods:**

Here we report a multi-epitope DNA vaccine targeted towards multiple subtypes of the influenza virus. The protective hemagglutinin (HA) antigens from H5/H7/H9 subtypes were screened for MHC II class-restricted epitopes overlapping with predicted B cell epitopes. We then constructed a DNA plasmid vaccine, pV-H3-EHA-H1, based on HA antigens from human influenza H3/H1 subtypes combined with the H5/H7/H9 subtype Th/B epitope box.

**Results:**

Epitope-specific IFN-γ ELISpot responses were significantly higher in the multi-epitope DNA group than in other vaccine and control groups (*P *< 0.05). The multi-epitope group significantly enhanced Th2 cell responses as determined by cytokine assays. The survival rate of mice given the multi-epitope vaccine was the highest among the vaccine groups, but it was not significantly different compared to those given single antigen expressing pV-H1HA1 vaccine and dual antigen expressing pV-H3-H1 vaccine (*P *> 0.05). No measurable virus titers were detected in the lungs of the multi-epitope immunized group. The unique multi-epitope DNA vaccine enhanced virus-specific antibody and cellular immunity as well as conferred complete protection against lethal challenge with A/New Caledonia/20/99 (H1N1) influenza strain in mice.

**Conclusions:**

This approach may be a promising strategy for developing a universal influenza vaccine to prevent multiple subtypes of influenza virus and to induce long-term protective immune against cross-species transmission.

## Background

Over the years, influenza has become a serious public health problem. With the potential for sudden outbreaks, rapid spread, and high incidence of complications, the prevalence of influenza infections has caused tremendous loss of human life and material resources [1,2]. Thus, it is important to develop new approaches towards preventing seasonal infections as well as potential pandemics of influenza.

Based on their internal protein antigens, different influenza viruses can be divided into 3 types: A, B, or C. The surface antigens, hemagglutinin (HA) and neuraminidase (NA) are also used to identify different subtypes. At present, the prevalent human influenza viruses are the type A H3/H1 and type B viruses. However, in recent years, multiple subtypes (H5/H7/H9) of the avian influenza virus (AIV) have been able to cross the species barrier to infect humans [3,4]. Around the world, the highly pathogenic avian influenza virus subtype H5N1 has caused infectious outbreaks in various human populations [5]. Influenza vaccines based on the conventional subtypes of each species have been unable to effectively prevent this rising trend. Creating vaccines which can provide long-term protection against more than one subtype of influenza has become a hot topic in vaccine development. However, due to the rapidly changing influenza virus or the phenomena of "antigenic shift" and "antigenic drift", developing a vaccine that can protect against all possible circulating viruses is extremely challenging.

Immunogenic epitopes in an antigen is determined by the major histocompatibility complex (MHC) class I for cytotoxic T cell lymhocytes (CTL) and MHC class II for T helper (Th) cells. These polymorphic MHC molecules present short peptides that are processed after an exogenous antigen (such as a viral protein) is taken up by antigen presenting cells (APC) such as macrophages and dendritic cells. These APC then "present" the peptide to the immune cells that recognize the MHC/peptide complex via the T cell receptor (TCR) or B cell receptor (BCR). Theoretically, given any set of MHC II restricted peptides presented to the Th cells, the optimal sequence would be those that could also stimulate B cells to produce antibodies since activation of antigen-specific Th cells also promote antibody production. By understanding the specific epitopes from pathogens that can stimulate optimal immune responses, we will better understand how to tailor vaccines to a specific population and/or pathogen.

Indeed, many studies have shown the efficacy of peptide-based vaccines in animal models [6], as well as in clinical studies against infectious diseases, including malaria [7,8], hepatitis B [9] and HIV-1 [10,11]. Development of an epitope-based vaccine for influenza may also be a useful strategy to overcoming the challenge of inducing a specific immune response against this constantly evolving virus. CTL epitopes mediate cytolytic effects on infected cells and induce inflammatory factors during viral clearance, while B cell epitopes can induce protective antibody-mediated humoral immune responses. Th epitopes can activate CD4+ T cells to carry out important immune regulatory functions, and the identification of specific epitopes derived from influenza virus has significantly advanced the development of peptide-based vaccines [12-15]. Improved understanding of the molecular basis of antigen recognition and human leukocyte antigen (HLA) binding motifs has allowed the development of rationally designed vaccines based on motifs predicted to bind to human class I or class II MHC. Therefore, identification of the corresponding functional influenza epitopes will have important theoretical and practical value in studies on immunity against virus infection and on vaccine development.

Presently, standard inactivated vaccines based on one or a few circulating strains are mainly utilized for prevention of influenza infection, but they cannot effectively deal with the current trend of increasing variations of the circulating viruses. A new influenza vaccine that can afford long-term and cross-species protection against multiple subtypes of influenza is imperative. Developing DNA vaccines that can stimulate both humoral and cellular immunity is a promising area of research. In particular, a multi-epitope DNA vaccine which expresses antigen genes in tandem can efficiently present the defined protective epitopes to stimulate the immune system while eliminating non-essential components or potential toxic fragments of traditional inactivated vaccines. Additionally, the development of such multivalent vaccines can be combined with other vaccine antigens to enhance immunogenicity. The advantage of combination vaccines is that they can potentially provide broader coverage to protect against rapidly mutating viruses such as influenza.

We report here the generation and evaluation of the immunogenicity of a DNA vaccine expressing HA based on human influenza H3/H1 combined with a class II MHC multi-epitope antigen (hereafter referred to as the "multi-epitope" vaccine). The vaccine was evaluated for induction of humoral and cellular immune responses in a mice model as well as for the protective efficacy against lethal H1N1 subtype virus challenge. We expected the vaccine targeted towards human influenza subtype H3 and H1 to provide total protection against these strains while at the same time achieving some level of protective efficacy against other influenza subtypes. This approach may be effective against rapidly mutating influenza and provide longer-term protection while laying the foundation for development of a new universal influenza vaccine.

## Materials and methods

### Mice, viruses and cells

Female BALB/c mice (6-8 weeks old) were used for immunization and challenge studies. All mice were maintained with free access to sterile food and water.

A/New Caledonia/20/99 influenza virus (H1N1) (GenBank CY033622) and A/Wisconsin/67/2005 (H3N2) strains were stored in the laboratory. Virus stocks were propagated in the allantoic cavity of 10-day-old embryonated chicken eggs for 48 h at 37°C. The viruses were titrated by the Reed and Muench method to determine the median lethal dose (LD_50_). Baby hamster kidney (BHK-21) cells were used for transient expression experiments. All experiments with influenza viruses were conducted under BSL-3 containment, including work in animals.

### Design of epitopes box and synthetic peptides

The HA gene sequences of the influenza H5, H7, H9 subtypes which have crossed species barriers to infect mammals and become vaccine strains were downloaded from NCBI http://www.ncbi.nlm.nih.gov with the following main reference sequence accession numbers, respectively: ISDN125873 (A/Indonesia/5/05(H5N1)), AAR02636 [A/Netherlands/127/03(H7N7)], and DQ997437 [A/swine/Shandong/nc/2005(H9N2)]. MHC II restricted epitopes were predicted bioinformatically by the network server SYFPEITHI and Multipre, and B cell epitopes were predicted using the network server BCEPRED http://www.imtech.res.in/raghava/bcepred/ or the Biomolecule simulation software Insight II (Accelrys, 2005). Th cell epitope predictions were based upon their cumulative binding affinity to six of the most common HLA-DRß1 alleles (DRß1*0101, DRß1*0301, DRß1*0401, DRß1*0701, DRß1*1101, and DRß1*1501). The network server BCEPRED was used for linear B cell epitope prediction which screens sequences based on hydrophilicity, accessibility, flexibility, antigenicity, polarity, and exposed surface residues. The Th epitope prediction was narrowed down to include as much as possible the epitopes which overlapped with the predicted B cell epitopes in order to obtain epitopes with dual functions of stimulating both T and B cells.

The Th/B cell epitope box was designed with "GPGPG" linkers between each epitope in order to reduce interference between epitopes and to ensure the proper processing and function of each epitope independently. The "KK" linker was also added to prevent the epitopes between subtypes from "splitting", that is, to avoid generation of new junctional epitopes [16,17]. Based on the design of the epitope box, the nucleotide sequences were codon-optimized and the peptides synthesized accordingly (Xu Guan Biological Engineering Co., Ltd. Shanghai, PR China). Peptides were dissolved in 20% DMSO and frozen at -80°C until use.

### Construction of plasmids

The HA genes of influenza A/New Caledonia/20/99 [H1N1] and A/Wisconsin/67/2005 [H3N2] were obtained by RT-PCR amplification of the isolated RNA. The H3HA, H1HA1 and the epitope box (termed EHA) sequences were inserted into the pMD18-T vector after addition of restriction sites *Nhe *I/*Hind *III, *Cla *I, *Xho *I, *Cla *I/*Xho *I and *Hind *III/*Xho *I, respectively, to yield pMD18-H3HA, pMD18-H1HA1 and pMD18-EHA. A eukaryotic expression vector, pVAX1 (Invitrogen, Carlsbad, CA, USA) was used to construct the following DNA vaccine vectors: pV-H3HA, pV-H1HA1, pV-H3-H1 and pV-H3-EHA-H1. The four DNA constructs were sequenced to confirm cloning accuracy before amplification in *Escherichia coli *JM109 and purification using endotoxin-free kits (QIAGEN, Valencia, CA). The final DNA preparations were resuspended in sterile saline solution and stored at -20°C until further use.

### Indirect immunofluorescence assay

BHK-21 cells were transfected with purified DNA from pV-H3HA, pV-H1HA1, pV-H3-H1, pV-H3-EHA-H1 and pVAX1 using Lipofectamine 2000 (Invitrogen) according to the manufacturer's protocol. In brief, cell monolayers were grown on glass coverslips in a 6-well plate and were then transfected with the plasmid DNA (10 μg/well). At 48 h after transfection, the cells were fixed with 0.05% glutaraldehyde and permeabilized with 0.5% Triton X-100 in phosphate-buffered saline (PBS), followed by incubation with rabbit anti-HA of A/New Caledonia/20/99 (H1N1), A/Wisconsin/67/2005 (H3N2), A/Indonesia/5/05(H5N1), A/Netherlands/127/03(H7N7), A/swine/Shandong/nc/2005(H9N2) polyclonal antibody [1:200 in poly(butylene succinate-co-terephthalate) (PBST)] for 1 h at 37°C. Fluorescein isothiocyanate (FITC)-conjugated goat anti-rabbit IgG antibodies [in PBS/bovine serum albumin (BSA)] were added and then incubated for 1 h at room temperature. After mounting the samples, fluorescence images were scanned using an Olympus microscope (BX51; Olympus, Japan).

### Immunization and virus challenge

In challenge experiments, four DNA vaccine groups and an empty vector pVAX1 control group of female Balb/c mice (n = 15) were immunized intramuscularly (IM, each with 100 μg of plasmid DNA in 100 μL of PBS [pH 7.4] in the two hind quadriceps). The immunization schedule consisted of 2 administrations with a 3-week interval, and bleeding was performed at 0, 1, 2, 3, 4 and 5 weeks after immunization for determination of antibody titers. To assess the efficacy of the cross-protective immunity of the 2 vaccine doses against lethal challenge 2 weeks after the second immunization, the immunized mice were anesthetized and intranasally challenged with 10 LD_50 _(50% lethal doses) of the A/New Caledonia/20/99 H1N1 virus in a final volume of 100 μL. The challenge experiments were performed in a biosafety level 3 (BSL3) facility (Military Veterinary Institute, Changchun, PR China).

### Viral lung titer measurements

To determine tissue viral titers, the lungs of surviving mice challenged with H1N1 were collected and homogenized by mechanical disruption. The viral titers were determined by plaque formation assay performed in MDCK cells in the presence of trypsin as previously described [18,19].

### Serum cytokine assays

A pre-coated enzyme-linked immunosorbent assay (ELISA) kit was used (Dakewe Biotech, PR China) to determine the cytokines levels of interferon (IFN)-γ and interleukin (IL)-4 in the immunized the mice according to the manufacturer's instructions. Serum samples (100 μl) from different groups of mice were tested in duplicate. After 36 h of incubation with the standards and samples, the plates were washed, followed by addition of 50 μl of the Streptavidin-HRP solution. The plates were incubated at 37°C for 60 min before washing again for at least five times, with a 1-2 min interval in between each wash. The diluted substrate was added at 50 μl per well and incubated at 37°C for 15 min. Finally, 50 μl of stop solution were added per well to terminate the reaction. Absorbance values were measured at 450 nm. Standard curves were drawn according to the instructions of the kits. The cytokines levels in the samples were calculated accordingly, expressed as ΔX ± SD, and differences between groups were analyzed statistically.

### IFN- γ ELISpot assays

The frequencies of IFN-γ secreting splenocytes were analyzed using a commercially available mouse IFN-γ pre-coated ELISpot assay according to the instructions of the manufacturer (Dakewe Biotech, PR China). Lymphocytes from the spleen were removed aseptically 10 days after a boost immunization, and a single cell suspension (10^6 ^cells/well) was prepared and stimulated with 20 μg/ml of the inactivated whole virus antigen preparations of A/New Caledonia/20/99 (H1N1) and A/Wisconsin/67/2005 (H3N2) or the following HA antigen peptides (20 μg/ml) of A/Indonesia/5/05(H5N1), A/Netherlands/127/03(H7N7), and A/swine/Shandong/nc/2005(H9N2): H5HA_141-155_, H5HA_206-223_, H5HA_302-316_, H7HA_165-181_, H7HA_255-269_, H7HA_182-196_, H9HA_123-140_, H9HA_73-90_, H9HA_37-54_. The plates were placed in a CO_2 _incubator at 37°C. The following day, the splenocytes were discarded, and the plates were extensively washed with pre-chilled PBS. IFN-γ spots were detected by a biotinylated anti-mouse IFN-γ specific antibody, followed by addition of streptavidin-horseradish peroxidase (HRP) and development with 3-amino-9-ethylcarbazole (AEC) substrate solution. The spots were counted using an automated ELISpot reader. The results were expressed as the number of spot-forming cells (SFC)/10^6 ^spleen cells. *P*-values were calculated using a permutation test stratified for the experiment.

### Antibody detection

Virus antigen specific serum antibodies were detected by ELISA. The inactivated H1N1 and H3N2 virus (50 ng/well) or standard antigens of H5, H7 and H9 subtype were coated overnight in 96-well plates (Costar, Cambridge, MA, USA). Following blocking of non-specific binding, the serum samples were diluted 100 times in PBS containing 0.5% (wt/vol) gelatin, 0.15% Tween 20, and 4% calf serum (ELISA diluent) and applied in duplicate wells for a 1 h incubation at 37°C. The plates were washed five times with PBS and then reacted with a 1:2000 dilution of HRP-labeled goat anti-mouse IgG (Zhongshan Goldenbridge Biotech) for 1 h at 37°C. After another five washes with PBS, the substrate was added (10 mg ortho-phenylenediamine [OPD] + 20 mL 0.015% hydrogen peroxide in phosphate/citrate buffer). After incubation for 15 min at 37°C, the reactions were terminated with 2N H_2_SO_4_. Subsequently, the absorbance values were determined at 492 nm using a Sunrise automated plate spectrophotometer and analyzed with Microsoft Excel 2007 for Windows. *P*-values were calculated to detect significant differences among the groups.

### Statistical analysis

The Lifetest procedure using the Kaplan-Meyer method and log rank test were applied for survival analyses between study groups (H1N1 survival study). All tests applied were two-tailed, and *P*-values of 5% or less were considered statistically significant. The data was analyzed using the SPSS Version 16.0 software.

## Results

### Selection of epitopes

**Figure 1:**
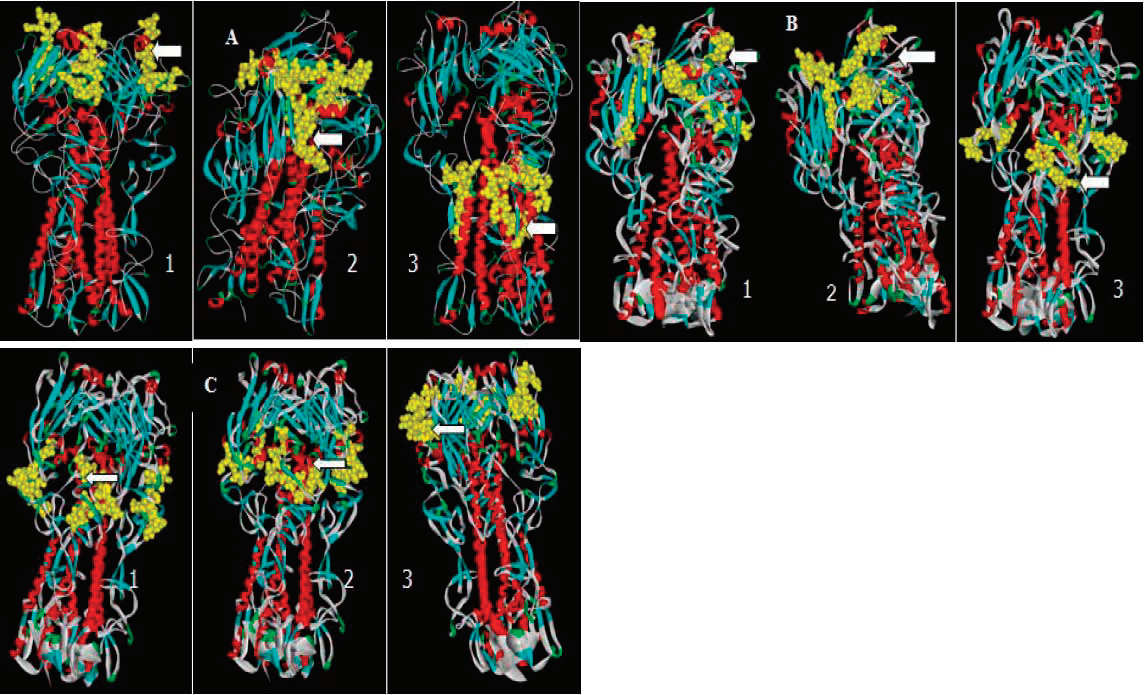
**Insight II software simulation of trimeric HA molecules on the surface of the virus**. The yellow area indicates the candidate epitope that mainly consists of random coiled structures and turns and may contain a small portion of a β-sheet structure or α-helix structure. Most of the candidate epitopes are exposed on the surface. (A) Simulated conformation of H5 HA candidate epitopes 1. HA_141~155 _2. HA_206~223 _3. HA_302~316 _(B) Simulated conformation of H7 HA candidate epitopes. 1. HA_165~181 _2. HA_182~196 _3. HA_255~269_. (C) Simulated conformation of H9 HA candidate epitopes. 1. HA_37~54 _2. HA_73~90 _3. HA_123~140._

SYFPEITHI and Multipre were used in different algorithms to predict Th epitopes. BCEPRED is an improved linear B cell epitope prediction method that utilizes multi-parameter analysis to predict potential B cell epitopes. Comprehensive analyses of both Th and B cell epitopes were performed to obtain a set of epitopes in which the predicted Th epitopes would also contain potential B cell epitopes. The selected epitope regions were re-evaluated for their spatial conformation and specificity to determine the final epitopes (Figure 1A-C). A final total of 9 Th and B cell epitopes were obtained for the H5, H7 and H9 subtypes of influenza (Table 1), and the corresponding peptides were synthesized with BSA conjugated at the C terminus.

**Table 1:** Predicted Th and B cell epitopes included in the multi-epitope vaccine

Epitope	Amino acid sequences
H5HA_141-155_	PSFFRNVVWLIKKNS
H5HA_206-223_	TLNQRLVPKIATRSKVNG
H5HA_302-316_	CPKYVKSNRLVLATG
H7HA_165-181_	DPALIIWGIHHSGSTA
H7HA_255-269_	SMGIQSDVQVDANCE
H7HA_182-196_	QTKLYGSGSKLITVG
H9HA_123-140_	NVSYSGTSKACSDSFYRS
H9HA_73-90_	GGKWSYIVERPSAVNGMC
H9HA_37-54_	HNGMLCATNLGHPLILNT

### Construction of the expression plasmid and immunogenicity assay

**Figure 2:**
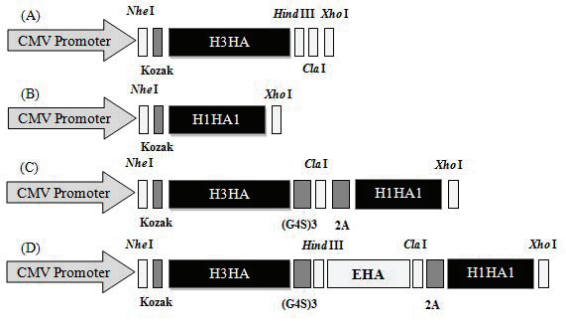
**Schematic diagram of the four DNA vaccine constructs**. (A) pV-H3HA, (B) pV-H1HA1, (C) pV-H3-H1, (D) pV-H3-EHA-H1. The Kozak sequence was added before the ORF to promote protein expression. The MHC class II-restricted epitope box, EHA, was inserted into the co-expression plasmid pV-H3-H1. A flexible linker (G4S)_3 _was added to allow effective fusion gene expression and promote the correct folding of expressed proteins. The autocleaving 2A protein linker from foot and mouth disease virus was also added to allow cleavage of the fusion protein after expression.

**Figure 3:**
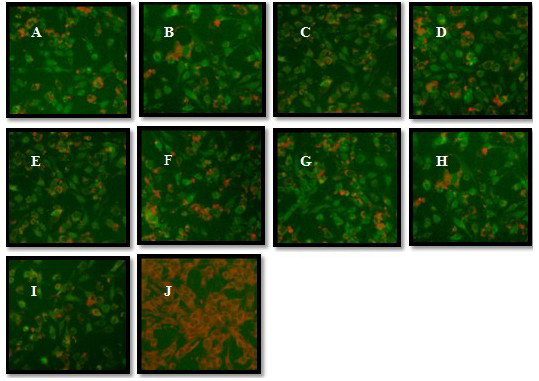
**Immunofluorescence assays to detect antigens expressed from the HA-based DNA vaccine by immune sera**. (A) pV-H3HA detected by H3+ serum, (B) pV-H1HA1 detected by H1+ serum, (C) pV-H3-H1 detected by H3+ serum, (D) pV-H3-H1 detected by H1+ serum, (E) pV-H3-EHA-H1 detected by H3+ serum, (F) pV-H3-EHA-H1 detected by H1+ serum, (G) pV-H3-EHA-H1 detected by H5+ serum. (H) pV-H3-EHA-H1 detected by H7+ serum, (I) pV-H3-EHA-H1 detected by H9+ serum, (J) pVAX1 control group. The BHK cells transfected with recombinant plasmids displayed specific fluorescence at the cell membrane and throughout the cytoplasm, while those transfected with the empty pVAX1 vector control were negative.

Before testing the immunogenicity of the vaccines, the four DNA vaccine constructs (Figure 2) were confirmed by sequencing. Protein expression from these constructs were also verified by transfection of pV-H3HA, pV-H1HA1, pV-H3-H1, pV-H3-EHA-H1 and pVAX1 (empty vector control) in BHK-21 cells, and the HA antigens and epitopes were detected by an immunofluorescence assay with HA anti-serum at 48 h post-transfection (Figure 3). The results indicated that the pV-H3HA, pV-H1HA1, pV-H3-H1, pV-H3-EHA-H1 plasmids could successfully expressed their corresponding proteins and multi-epitopes, thereby validating the use of the plasmids in subsequent experiments.

### Analysis of cytokine levels

**Figure 4:**
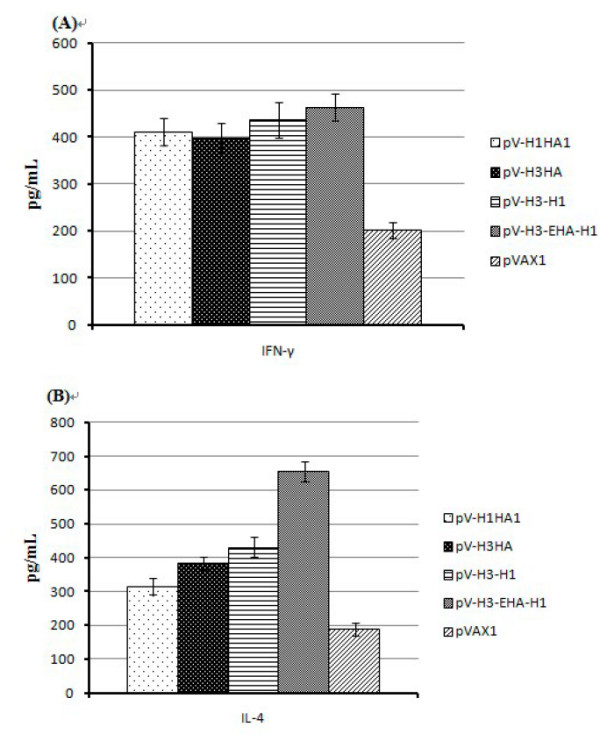
**IFN-γ (Th1) and IL-4 (Th2) cytokine levels in serum samples**. (A) IFN-γ secretions of the immunized groups were significant higher than that of the control pVAX1 group (*P *< 0.01) but were not significantly different from each other (*P *> 0.05). (B) The IL-4 level of the multi-epitope vaccine group was significantly higher than the other 3 groups (*P *< 0.05). These data represent 3 repeated cytokine measurements.

Fourteen days after the boost immunization, the sera were collected and analyzed for IFN-γ and IL-4 levels levels by ELISA (Figure 4A, B). The order of the IFN-γ levels detected in the immune sera were as follows: multi-epitope immune group (pV-H3-EHA-H1, 463) > two-subtype co-expression immune group (pV-H3-H1, 435) > H1 group (pV-H1HA1, 410) > H3 group (pV-H3HA, 398) > pVAX1 control group (201). The serum IFN-γ levels of the immunized groups were significantly higher (*P *< 0.01) than that of the control group, indicating that all of the vaccines tested effectively stimulated Th1 type responses. The multi-epitope group displayed the highest level of IFN-γ secretion, although the difference was not significant compared with the other 3 groups (*P *> 0.05).

As for the detection of IL-4 levels in the immune sera, the order was determined as follows: multi-epitopes immune group (pV-H3-EHA-H1, 654) > two-subtype co-expression immune group (pV-H3-H1, 431) > H3 immune group (pV-H3HA, 383) > H1 immune group (pV-H1HA1, 315) > pVAX1 control group (188). The IL-4 levels of the immune groups were also significantly higher (*P *< 0.01) than that of the pVAX1 control group. The IL-4 levels in the serum of the multi-epitopes group were significantly higher than that of the other immune groups (*P *< 0.05), indicating that the immunized groups had significantly enhanced Th2 cell function. Combined with the analysis of IFN-γ levels above, these findings demonstrated that the multi-epitope vaccine induced the greatest levels of vaccine specific immune responses in mice, and the use of these epitopes tended to produce Th2 cytokines and promoted humoral immunity.

### Cell-mediated immune responses induced in DNA plasmid immunized mice

**Figure 5:**
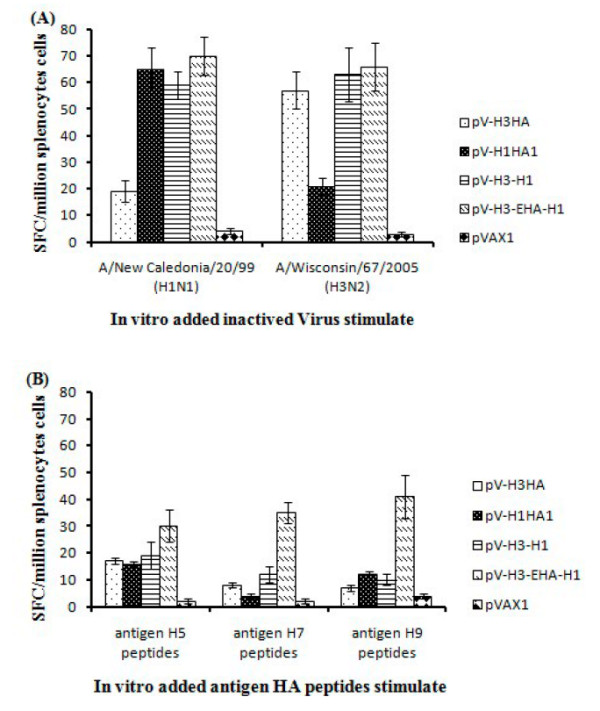
**Cellular immune responses in vaccinated mice**. Specific responses of splenocytes taken 14 days after the second boost were determined by the IFN-γ ELISpot assay with stimulation from (A) A/New Caledonia/20/99 (H1N1) and A/Wisconsin/67/2005 (H3N2) inactivated whole virus, and (B) influenza H5, H7 and H9 subtype specific peptides. Upon stimulation with the H7 and H9 subtype epitope peptides, the splenocytes from the multi-epitope DNA vaccine pV-H3-EHA-H1 showed significantly higher responses than other immunized groups (*P *< 0.05). Data are presented as mean ± SD of five mice per group. SFC, spot forming cells.

To evaluate the cellular responses to vaccination, splenocytes were harvested from five immunized mice from each group at 35 days after vaccination. Representative data from three repeated ELISpot assays detecting IFN-γ secretion from virus or peptide stimulated splenocytes are shown in Figure 5. Significant IFN-γ responses were observed in the immunized group as compared to cells from non-immunized mice following *in vitro *incubation with whole inactivated viruses [influenza virus A/Wisconsin/67/2005 (H3N2) and influenza virus A/New Caledonia/20/99 (H1N1)] (Figure 5A). The multi-epitope DNA group (pV-H3-EHA-H1) produced the most spots under the stimulations described above, but the differences were not significant (*P *> 0.05) compared to other immunized groups. However, the multi-epitope DNA group did have significantly (*P *< 0.05) higher levels of IFN-γ secretion than the other groups in response to peptide antigens (Figure 5B).

### Antibody responses induced in DNA vaccine immunized mice

**Figure 6:**
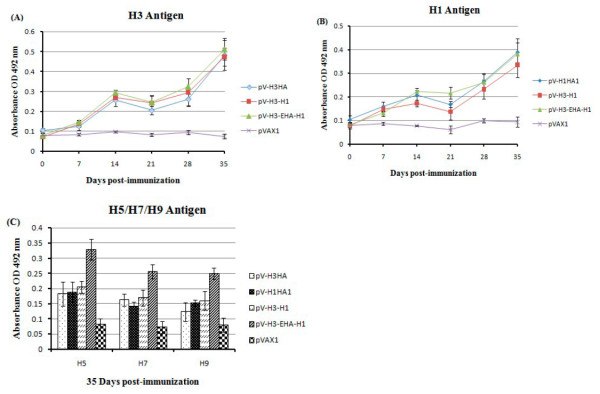
**Humoral immune responses in vaccinated mice**. ELISAs to detect virus-specific IgG antibodies were performed utilizing plates coated with (A) A/Wisconsin/67/2005 (H3N2), (B) A/New Caledonia/20/99 (H1N1), or (C) influenza standard antigens of H5, H7, H9 subtypes. Data shown are mean antibody titers of five mice in each group with coefficients of variations (error bars). The differences in titers between experimental groups and the negative control group (pVAX1) were statistically significant (*P *< 0.01). However, the differences in the responses against the H3N2 and H1N1 antigens in the ELISA between the multi-epitope pV-H3-EHA-H1 and pV-H3 and between pV-H1 and pV-H3-H1 groups were not significant (*P *> 0.05). With the H5, H7 and H9 standard antigens as the coated proteins in the ELISA, the responses in the pV-H3-EHA-H1 group were significantly higher than those of the other vaccine groups at 35 DPI (*P *< 0.05).

In evaluating the development of virus-specific IgG against the H3 and H1 subtypes of influenza by ELISA (Figure 6A, B), the antibodies were detectable from the first week after immunization, rising after the second week, and then decreased slightly from the third week after a rapid increase to its peak. At 35 days post-inoculation (DPI), virus specific antibody levels in all immunization groups were significantly higher than that in the control group (*P *< 0.01), but the levels of the different vaccine groups were not significantly different from each other (*P *> 0.05). That is, the IgG antibody levels induced were equivalent between the multi-epitope vaccine group (pVAX1-H3-EHA-H1), the single antigen expressing groups (pV-H3HA and pV-H1HA1) and the dual antigen expressing group (pV-H3-H1).

From the analysis of H5, H7, H9 subtypes of influenza virus-specific IgG antibodies (Figure 6C), the various vaccine groups generated significantly higher antibody levels than the control group in the ELISA (*P *< 0.01) at 35 DPI. Furthermore, the Th/B multi-epitope group (pV-H3-EHA-H1) had significantly higher H5, H7 and H9 subtype IgG levels than the other immunized groups (pV-H3HA, pV-H1HA1 and pV-H3-H1), suggesting that the selected epitopes could effectively stimulate virus-specific antibodies. The highest antibody levels detected by ELISA were against the H5 epitopes, suggesting that this vaccine would theoretically be more effective against this virus subtype in mice.

### Protection against lethal dose challenge with influenza H1N1

**Figure 7:**
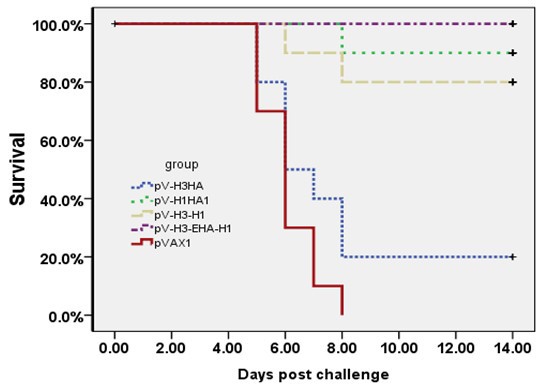
**Survival of immunized mice from lethal dose challenge of H1N1 virus**. Groups of 10 mice were immunized IM with the recombinant vaccines (striped lines) or with the pVAX1 vector control (solid line). The schedule of administration was 2 times with a 3-week interval, and the challenge with 10 LD_50 _of influenza virus (A/New Caledonia/20/99 (H1N1)) was given 2 weeks after the boost. A significant difference (*P *< 0.05) in survival was observed between multi-epitope group pV-H3-EHA-H1 (100%) and pV-H3 (20%), but no significant difference was found compared to the pV-H1HA1 (90%) and pV-H3-H1 (80%) groups (*P *> 0.05).

To test the efficacy of the vaccines, BALB/c mice (6 weeks) were immunized IM with 200 μg of each vaccine and challenged with 10 LD_50 _of A/New Caledonia/20/99 (H1N1) influenza strain. Their survival rates were monitored for the following 14 days. The mice began to show clinical signs or death from influenza infection on day 5 post-challenge. Figure 6 shows the survival curve following these immunizations, and the final survival rates of the pV-H1HA1, pV-H3-H1, and pV-H3-EHA-H1 immunized mice were 90%, 80%, and 100%, which were significantly higher (*P *< 0.01) than that of the pV-H3HA and pVAX1 control groups (20% and 0%, respectively; Figure 7). Immunization with the multi-epitope vaccine resulted in complete protection against the lethal dose virus challenge, which was better than the single expression (pV-H1HA1) and co-expression immunized group (pV-H3-H1).

### Lung viral titers

**Figure 8:**
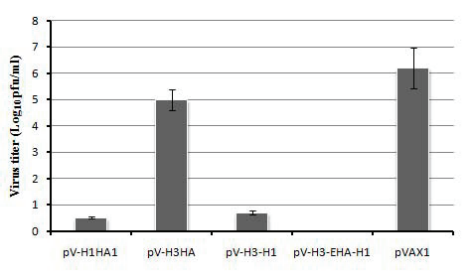
**Viral lung titers in immunized animals**. Five weeks after immunization with pV-H3-EHA-H1, pV-H3HA, pV-H3-H1, pV-H1HA1 or pVAX1 control, protection of mice from intranasal challenge with 10 LD_50 _A/New Caledonia/20/99 (H1N1) was determined by detection of viral titers in the lung using a plaque formation assay on MDCK cells. Log_10 _values of the mean titer for each group are shown.

The lungs were harvested from mice which survived the viral challenge, and viral titers were determined by plaque formation assays in MDCK cells. Because almost all the mice which received the pVAX1 control and pV-H3HA immunizations did not survive, lungs from dead mice in these groups had to be selected to test for viral titers. As expected, the mice of the pVAX1 control group and pV-H3HA group all had positive viral titers in the lung. By contrast, no measurable virus titers were detected in the lungs in the multi-epitope immunized group, and somewhat lower levels of virus (expressed as plaque forming units, or PFU) were observed in the pV-H3-H1 and pV-H1HA1 immunized groups (Figure 8).

## Discussion

In light of the recent 2009 H1N1 pandemic, there is an urgent need to develop new influenza vaccines. New influenza vaccines should have the following characteristics: low cost, high level immunogenicity, rapid preparation, protection against rapid virus mutation and long-term protection against multiple subtypes of influenza, especially against potential influenza pandemic strains.

The purpose of this study was to further develop and evaluate a novel approach to vaccination based on multi-subtype influenza epitopes using mice as a mammalian model. We assessed the immunogenicity of an H3/H1-derived multi-epitope DNA vaccine and its protective efficacy against H1N1 virus challenge. The experiment was set up to systematically compare the specially designed multi-epitope vaccine with the separate antigen components of the immunized groups and a control group.

The MHC II molecule pathway of antigen processing is first activated in an APC by phagocytosis, pinocytosis, or receptor-mediated endocytosis of an exogenous antigen. The phagocytic lysosome products are digested into linear epitopes and then later associated with MHC II molecules to be presented on the surface of the APC. Th cells and APC recognition between cells and signal transduction and the resulting induced Th cell activation play an important role in the initiation of an acquired immune response, maintenance of responses in chronic infections and development of immune memory. Activated Th cells produce cytokines that can effectively regulate cytotoxic T cells, B cells and phagocytic cell functions [20].

B cell epitopes form the basis of humoral immunity in that they determine the specificity of antibodies. B cells can capture antigens through the BCR and function as APC to activate Th cells. Activated Th cells can also activate B cells in turn to produce antibodies against the corresponding antigen. An ideal immunogenic epitope is one that elicits responses from both Th and B cells [21]. Therefore, the purpose of this study was to design a vaccine with a minimal set of epitopes that are predicted to cross-stimulate both Th and B cell subsets.

IFN-γ is the defining Th1 type cytokine, with important immunoregulatory functions including the ability to activate macrophages, induce monocyte cytokine secretion, affect the body's Th1/Th2 balance, regulate antigen presenting cells, and significantly increase MHI-1 and MHC-II molecule expression [22]. IL-4 is the representative Th2 type cytokine, with the ability to promote B cell proliferation and antibody production [23]. In this study, we examined these two cytokines to evaluate the immune bias induced by the multi-epitope vaccine. Our analyses revealed that the IFN-γ level of the multi-epitope vaccine immunized group was the highest, indicating effective stimulation of the Th1 response, although it was not significantly different from the other three vaccine groups (*P *> 0.05). However, the stimulation of IL-4 levels by the multi-epitope vaccine was significantly higher than the other vaccine groups (*P *< 0.05), indicating that the multi-epitope pV-H3-EHA-H1 vaccine could enhance Th2 cell immune function. Therefore, the multi-epitope vaccine tended to induce a dominant Th2 response which could then promote humoral immune responses.

Production of antibodies against viral infection is an important effector function, and the level of humoral immunity reflects the ability and strength of the body to block infection, to rid itself of the virus if infection occurs, or at least to prevent tissue damage [24]. In perspective, the four DNA vaccines were effective in stimulating specific antibodies against the major antigens from the H3 and H1 subtypes of influenza in mice. Specific antibody levels increased with the number of immunizations, rapidly rising after the boost immunization. At 35 DPI, the multi-epitope immunization group had virus-specific antibody levels equivalent to the H1 and H3 immune groups, suggesting that the epitopes induced by the structural HA antigen is still a major determinant of specific antibody production. From analyzing the virus-specific IgG epitopes associated with H5, H7, H9 subtypes of influenza, the multi-epitope immunized group showed advantages compared with the pV-H1HA1, pV-H3HA and pV-H3-H1 groups (*P *< 0.05), which confirmed that epitopes in a multi-epitope vaccine could induce functional antibodies, especially those of the H5 subtype.

The DNA vaccines also effectively stimulated cellular immune responses in mice as evaluated by the IFN-γ ELISpot assays. For the H3 and H1 IFN-γ responses, the multi-epitope group produced the highest number of spots, but not significantly different compared with the single antigen expressing and the dual antigen expressing groups (*P *> 0.05). When stimulated with peptides associated with the H5, H7, H9 antigens, the splenocytes of the multi-epitope vaccine group produced the highest number of IFN-γ spots which was significantly different from that of the other groups. Together these results indicate that the HA subtype and the specific epitopes may play major roles in inducing Th1 cellular immune responses.

Further evidence of the efficacy of the multi-epitope vaccine was provided by the *in vivo *challenge studies. These protection experiments showed differences in the morbidity of the animals as well as a statistically significant difference between the survival curves of the vaccinated groups and the pVAX1 control groups. The multi-epitope group showed 100% protection against lethal challenge, but it was not significantly different compared to the pV-H1HA1 and pV-H3-H1 groups (*P *> 0.05). No measurable virus titers were detected in the lungs of the mice in the multi-epitope immunized group. Somewhat lower virus titers were observed in the pV-H3-H1 and pV-H1HA1 groups, which indicated that the constituent epitopes contributed to the cross-protective immunity against H1N1 viral challenge.

## Conclusion

Overall, the multi-epitope DNA vaccine induced significant levels of humoral and cellular responses as well as provided cross-protective immunity. This study demonstrates the proof-of-principle that a universal DNA vaccine with engineered epitopes may protect against multiple subtypes of influenza virus, afford long-term immune protection, and prevent cross-species transmission.

## Competing interests

The authors declare that they have no competing interests.

## Authors' contributions

LT performed most of the experimental work and drafted the manuscript. DZ, BH and ZYW participated in the analysis of humoral and cellular responses. MYT participated in the immunization of mice. NYJ and HJL revised the manuscript for important intellectual content and gave final approval of the version to be published. All authors read and approved the final manuscript.
